# The Global Transcriptional Response of Fission Yeast to Hydrogen Sulfide

**DOI:** 10.1371/journal.pone.0028275

**Published:** 2011-12-02

**Authors:** Xu Jia, Weizhi He, Alastair I. H. Murchie, Dongrong Chen

**Affiliations:** 1 Institute of Biomedical Science, Fudan University, Shanghai, China; 2 School of Pharmacy, Fudan University, Pudong, Shanghai, China; University of Louisville, United States of America

## Abstract

**Background:**

Hydrogen sulfide (H_2_S) is a newly identified member of the small family of gasotransmitters that are endogenous gaseous signaling molecules that have a fundamental role in human biology and disease. Although it is a relatively recent discovery and the mechanism of H_2_S activity is not completely understood, it is known to be involved in a number of cellular processes; H_2_S can affect ion channels, transcription factors and protein kinases in mammals.

**Methodology/Principal Findings:**

In this paper, we have used fission yeast as a model organism to study the global gene expression profile in response to H_2_S by microarray. We initially measured the genome-wide transcriptional response of fission yeast to H_2_S. Through the functional classification of genes whose expression profile changed in response to H_2_S, we found that H_2_S mainly influences genes that encode putative or known stress proteins, membrane transporters, cell cycle/meiotic proteins, transcription factors and respiration protein in the mitochondrion. Our analysis showed that there was a significant overlap between the genes affected by H_2_S and the stress response. We identified that the target genes of the MAPK pathway respond to H_2_S; we also identified that a number of transporters respond to H_2_S, these include sugar/carbohydrate transporters, ion transporters, and amino acid transporters. We found many mitochondrial genes to be down regulated upon H_2_S treatment and that H_2_S can reduce mitochondrial oxygen consumption.

**Conclusion/Significance:**

This study identifies potential molecular targets of the signaling molecule H_2_S in fission yeast and provides clues about the identity of homologues human proteins and will further the understanding of the cellular role of H_2_S in human diseases.

## Introduction

Hydrogen sulfide (H_2_S) is a gasotransmitter; a biologically active gaseous endogenous signaling molecule. The cellular effects of hydrogen sulfide have been linked to many of the body's physiological systems such as the cardiovascular system and central nervous systems. Consequently abnormal hydrogen sulfide metabolism is implicated in many diseases including hypertension, heart disease, atherosclerosis and inflammation [1], [2]. H_2_S has a number of molecular targets in cells. It is known that H_2_S interacts with the ATP-sensitive potassium (K_ATP_) channel and can relax blood vessel and smooth muscle cells through opening the K_ATP_ channel. Recent studies have shown that H_2_S can also act on other transmembrane proteins including Ca2+ channels, Cl- channels and the N-methyl-D-aspartic acid receptor [3]. H_2_S also effects the MAPK pathway through direct interactions with the cellular protein kinases P38 MAPK, ERK, Akt and P21 [2], [4]–[10]. Additionally, a number of transcription factors respond to H_2_S notably nuclear factor NF-kB, STAT3, Nrf-2 and Hif [1], [11]–[13]. In addition, H_2_S has a role in protecting gastric mucosal epithelial cells against oxidative stress [14]. Despite the wealth of accumulated data on the cellular effects of H_2_S, the molecular mechanism and cellular targets of H_2_S are far from completely understood.

Fission yeast *Schizosaccharomyces pombe* (*S. pombe*), as a model organism, has been widely used for the studies of many basic biological processes including control of the cell cycle and stress response. The availability of a well annotated whole genome sequence [15] allowed genome-wide studies of many fundamental questions by microarray. Microarray analysis of global gene expression profiles and regulatory mechanisms of environmental stress responses, cell cycle control and meiosis have been reported in fission yeast [16]–[18]. Here we have used fission yeast microarrays to identify cellular targets of H_2_S and to further understand the cellular mechanism of H_2_S action. In this paper, we report the change in the global gene expression profile response to H_2_S in *S. pombe* cells. Microarray analysis shows that there is a significant overlap between effects of H_2_S and the stress response. In response to H_2_S new down-stream genes in the MAPK pathway have been identified. We show that H_2_S causes differential expression of many transmembrane transporters and proteins involved in the cell cycle/meiosis. We have found that a significant number of mitochondrial genes are down regulated in response to H_2_S; leading to reduced mitochondrial oxygen consumption. We anticipate that this study would provide clues on molecular targets and signaling molecules of homologues human proteins.

## Materials and Methods

### Fission yeast Strains, media and techniques

The strain used in this study is wild type *S. pombe* 972*h^−^*. Media and culture condition of *S. pombe* were as described in [19]. 50 µM of NaHS (Sigma, St Louis, MO, USA) were used for treatment of wild type cells for 30 min at 30°C. The concentrations of NaHS selected in the present study did not affect the pH values of the culture medium and the sodium ion content in NaHS was negligible. The growth of the wild type *S. pombe* cells (starting from OD_600_ = 0.1, ∼2×10^6^cells/ml) was measured every two hours on the addition of 0, 100, 200, 300, 400 and 500 µM NaHS.

### RNA extraction for microarray analysis

The wild type *S. pombe* cells were grown in liquid medium to OD_600_ = 0.5 (∼1×10^7^cells/ml) and total RNA was extracted from cells using a hot phenol method as described in [20].

### RNA extraction and real-time PCR Analysis

Cells were grown in liquid medium to OD_600_ = 0.5 (∼1×10^7^cells/ml), total RNA were extracted using TRIzol Reagent (invitrogen) as manufacture required. Reverse transcription of RNA was performed (TaKaRaPrimeScipt™ 1st Strand cDNA Synthesis kit) followed by quantitative real-time PCR on iQ5 Continuous Fluorescence Detector System (Bio-Rad). The PCR reactions contained 250 nM of forward and reverse primers, 1 µl cDNA(5 ng), 10 µl 2× SYBR-green Real time PCR Master Mix (SYBR Premix Ex Taq™, TaKaRa) in a total volume of 20 µl. All results are generated from at least two independent biological repeats and for each biological experiment four technical repeats were performed.

### DNA microarray and data analysis

The GeneChip® Yeast Genome 2.0 Array from Affymetrix was used in this study. This array includes 5,021 probe sets for all 5,031 genes present in *S. pombe*. The microarray experiments including RNA purification, cDNA probe preparation, hybridization, washing, scanning, image analysis, normalization and data processing were performed by Shanghai Biochip Co., Ltd. as described in the Affymetrix GeneChip_Expression Analysis Technical Manual. Three biological repeats were performed for the microarray experiments. All experiments conformed to minimum information about a microarray (MIAME) guidelines and have been deposited in the GEO database (accession number GSE30025). Annotation of the genes represented on the microarray including “gene_ID” and “gene symbol” were obtained from NetAffxTM Analysis Center. For the identification of differentially expressed genes Significance Analysis of Microarrays (SAM) and fold change was used such that the False Discovery Rate (FDR)<0.05 and fold change>1.5 or 2. The Fisher test was used to evaluate the statistical significance (P value) of overlaps between two gene groups. Functional classification was accomplished by using DAVID [21] and cluster 3.0 [22].

### Measurement of mitochondrial oxygen consumption in *S. pombe*

Cell respiration Oxygen consumption by intact cells was measured as an indication of mitochondrial respiration activity. *S. pombe* cells were cultured at 30°C until OD_600_ = 0.5 (∼1×10^7^cells/ml) and then treated with 50 µM of NaHS for 30 min. The control sample was not treated with NaHS. Cells were harvested and 200 µl of cultures was placed in triplicate to a BD Oxygen Biosensor System plate (BD Biosciences, San Diego, CA, USA). Plates were sealed and measured on a fluorescence spectrometer (Molecular Devices, SpectraMax M5, CA, USA) at 1 min intervals for 60 min. at an excitation of 485 nm and emission of 630 nm. Oxygen consumption curves were made after the measurements and Vmax as the maximum oxygen consumption rate was calculated.

## Results and Discussion

### Determination of NaHS concentration for treatment of *S. pombe* cells

**Figure 1:**
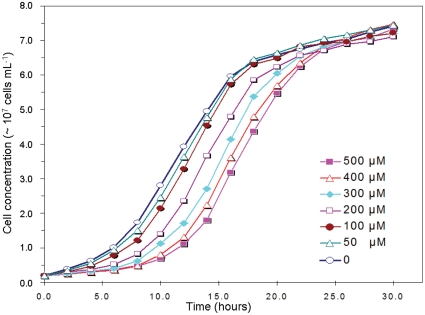
The growth curve of fission yeast cells on addition of NaHS. The wild type cells was grown in the presence of 0, 50 µM, 100 µM, 200 µM, 300 µM, 400 µM and 500 uM NaHS. Cell density was measured every 2 hours. The growth curve shows that addition of NaHS inhibits the growth of the wild type cells.

To determine the genome-wide response to hydrogen sulfide in *S. pombe* cells by microarray, we treated wild-type 972*h^−^* cells with the H_2_S precursor sodium hydrogen sulfide (NaHS). NaHS has been widely used as a donor of H_2_S to treat mammalian cells for investigation of the cellular effects of H_2_S. The concentration of NaHS used in some of these studies has ranged from 10–2000 µM [14], [23]–[25]. However, the effects of NaHS on S. pombe cells were not known. We therefore treated wild type *S. pombe* cells with 0, 50, 100, 200, 300, 400 and 500 µM NaHS and measured growth curves under this range of NaHS conditions. Figure 1 shows that NaHS inhibits *S. pombe* cells growth in a concentration dependant manner and that cells treated with different doses of NaHS all reach their maximum cell density after 24 h. We note that 50 µM NaHS causes only a slight reduction in growth of *S. pombe* cells. We also observed that high concentrations of NaHS (greater than 250 µM) change the pH of YE medium (data not shown). We therefore chose to treat *S. pombe* cells with 50 µM NaHS for 30 min. because these conditions do not cause death of *S. pombe* cells. This concentration of H_2_S does not change the pH of the media. The physiological concentration of H_2_S in various human tissues has been reported in this range, for example, in brain tissue H_2_S is estimated at 50–160 µM [26]–[28]. However, the true concentration of H_2_S in fission yeast cells under our experimental conditions remains unknown as techniques for the accurate measurement of intracellular H_2_S have not been developed.

### Overview of the microarray analysis

**Figure 2:**
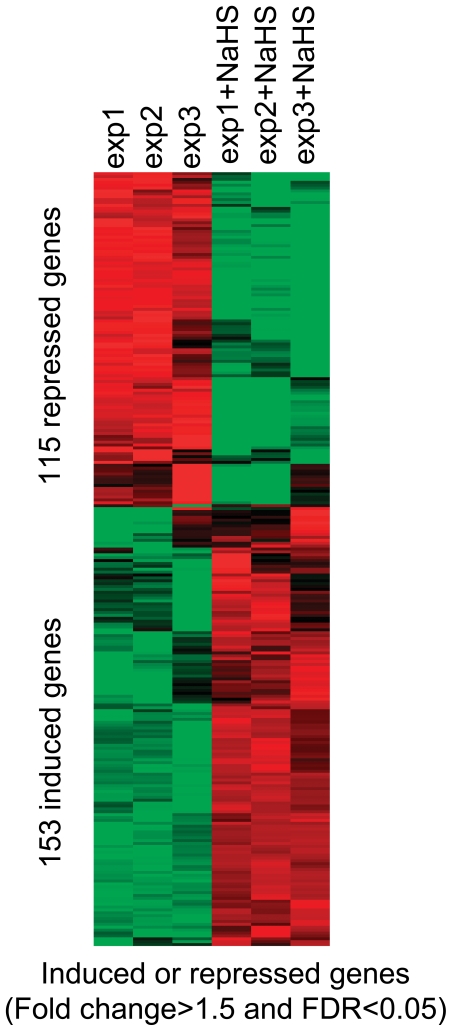
Hierarchical cluster of genes whose expression changed by more than 1.5 fold (FDR<0.05) in response to H_2_S with three biological repeats. The samples that are not treated with NaHS are labeled as exp1, exp2 and exp3 and samples that are treated with NaHS labeled as exp1+ NaHS, exp2+ NaHS and exp3+ NaHS. Note that in response to NaHS, 153 genes are induced and 115 genes are repressed.

We performed genome-wide expression analyses upon treatment of the wild-type 972*h^−^* cells with NaHS. The wild-type 972*h^−^* cells were treated with 50 µM NaHS for 30 min. Total RNA was extracted from untreated wild-type cells and from cells that had been treated with NaHS for DNA microarray hybridization. Each biological experiment was repeated three times. We combined the statistical analysis method (SAM) and relative fold changes to identify differentially expressed genes. SAM analysis showed that there were 720 genes with FDR<0.05. Table 1 is a list of genes whose expression level reached FDR<0.05 in SAM analysis and also was changed by 2 fold or greater in response to NaHS treatment. Table 1 shows genes with FDR<0.05, 63 genes induced more than 2 fold and 17 genes repressed more than 2 fold. We also identified genes whose expression level in SAM analysis FDR<0.05 and changed by 1.5 fold (Table S1). Table S1 shows genes with FDR<0.05, 153 genes were induced greater than 1.5 fold and 115 genes were repressed greater than 1.5 fold. Only those genes whose expression changes with an FDR<0.05 are discussed below. The magnitude of the gene expression response to NaHS treatment varied for induced or repressed genes: whereas the highest expression level for induced genes is 10 fold, the lowest expression level for repressed genes is 3 fold. We used cluster 3.0 [22] to cluster the differentially expressed genes that were induced or repressed by 1.5 fold (Figure 2). The cluster in Figure 2 shows the differentially expressed genes in the three biological replicates, revealing that the biological replicates were indeed similar and assembled into the closet branches of the cluster. This confirms the reproducibility of the biological replicates. To further confirm the microarray data, we randomly picked 8 genes from the genes whose expression level induced (4 genes) or repressed (4 genes) and performed real-time PCR and compared them to the microarray data. Table 2 shows that the 4 genes that are induced or repressed in the microarray are also induced or repressed in the real-time PCR experiment.

**Table 1:** Genes induced or repressed greater than 2 fold in response to H_2_S.

Gene symbol	Fold change	Regulation	Gene annotation
**Cellular response to stress**			
SPAC17G6.13	2.52	up	sequence orphan
SPCC338.18	4.49	up	sequence orphan
SPAC22H12.01c	2.55	up	sequence orphan
SPAC186.03///SPBPB21E7.09	2.33	up	L-asparaginase///L-asparaginase (predicted)
hri1	2.99	up	eIF2 alpha kinase Hri1
SPBP4H10.10	2.53	up	rhomboid family protease
SPAC11D3.17	2.44	up	zinc finger protein
ght3	4.49	up	hexose transporter Ght3 (PMID 10735857)
psi	2.12	up	DNAJ domain protein Psi1
SPAC27D7.09c	2.38	up	S. pombe specific But2 family protein
SPBC16D10.08c	2.16	up	heat shock protein Hsp104 (predicted)
SPBC1271.08c	6.80	up	sequence orphan
SPAC11D3.09	2.28	up	agmatinase (predicted)
SPAPJ691.02	2.02	up	yippee-like protein
SPCC191.01	2.16	up	sequence orphan
SPCC70.08c	2.67	up	methyltransferase (predicted)
ssa1	2.98	up	heat shock protein Ssa1
SPBPB2B2.12c	6.46	up	UDP-glucose 4-epimerase
ght4	5.46	up	hexose transporter Ght4 (PMID 10735857)
SPCC1739.08c	6.91	up	short chain dehydrogenase
SPAC1F8.04c	2.27	up	hydrolase (predicted)
isp7	2.07	up	2-OG-Fe(II) oxygenase superfamily protein
SPBC4F6.17c	2.51	up	mitochondrial matrix chaperone Hsp78 (predicted)
SPBC1271.07c	6.52	up	N-acetyltransferase (predicted)
SPBC1683.06c	5.45	up	uridine ribohydrolase (predicted)
SPBP26C9.01c	2.52	up	hydroxyacid dehydrogenase (predicted)
SPACUNK4.17	0.46	down	NAD binding dehydrogenase family protein
gut2	0.42	down	glycerol-3-phosphate dehydrogenase Gut2
SPBC3H7.02	0.41	down	sulfate transporter (predicted)
SPCC576.17c	0.44	down	membrane transporter
SPAC22F8.05	0.48	down	alpha,alpha-trehalose-phosphate synthase (predicted)
**Transport**			
**monosaccharide/carbohydrate/transmembrane/iron ion/iron ion transmembrane transport**			
ght1	2.02	up	hexose transporter Ght1 (PMID 10735857)
ght3	2.24	up	hexose transporter Ght3 (PMID 10735857)
ght4	2.25	up	hexose transporter Ght4 (PMID 10735857)
ght5	5.46	up	hexose transporter Ght5 (PMID 10735857)
ght6	8.64	up	hexose transporter Ght6 (PMID 10735857)
fip1	2.53	up	iron permease Fip1
fio1	2.17	up	iron transport multicopper oxidase Fio1
str3	4.49	up	siderophore-iron transporter Str3 (PMID 12888492)
SPCC285.05	2.51	up	purine nucleoside transporter (predicted)
SPBC4F6.17c	5.43	up	mitochondrial matrix chaperone Hsp78 (predicted)
SPBC3H7.02	0.44	down	sulfate transporter (predicted)
SPCC576.17c	0.38	down	membrane transporter
SPCC794.04c	0.41	down	membrane transporter
**amino acid transport**			
isp5	3.15	up	amino acid permease Isp5
SPBPB2B2.01	2.52	up	amino acid permease, unknown 12
SPAP7G5.06	10.19	up	amino acid permease, unknown 4
**Oxidation/reduction**			
SPCC1739.08c	6.91	up	short chain dehydrogenase
SPCC1223.09	2.75	up	uricase (predicted)
isp7	2.07	up	2-OG-Fe(II) oxygenase superfamily protein
SPCC794.01c	3.13	up	glucose-6-phosphate 1-dehydrogenase (predicted)
fio1	2.25	up	iron transport multicopper oxidase Fio1
SPAC1039.06	2.14	up	alanine racemase (predicted)
SPBP26C9.01c	2.52	up	hydroxyacid dehydrogenase (predicted)
gut2	0.42	down	glycerol-3-phosphate dehydrogenase Gut2
SPAC3A11.07	0.48	down	NADH dehydrogenase
cad1	0.39	down	sulfide-quinone oxidoreductase
**Meiosis/M phase of meiotic cell cycle/meiotic cell cycle**			
SPAC17G6.13	2.52	up	sequence orphan
SPAC11D3.03c	4.91	up	meiotic chromosome segregation protein
ght6	2.02	up	hexose transporter Ght6 (PMID 10735857)
ste7	2.57	up	meiotic suppressor protein Ste7
SPAC22H12.01c	2.55	up	sequence orphan
SPBC359.06	5.86	up	adducin
rem1	2.14	up	meiosis-specific cyclin Rem1
meu10	2.76	up	conserved fungal family
nep2	3.68	up	nedd8 protease Nep2
SPBC106.08c	0.49	down	DUF1773 family protein 1
SPACUNK4.19	0.43	down	sequence orphan
**Cellular amide metabolic process**			
car1	2.34	up	arginase Car1 (PMID 7985419)
SPCC794.01c	3.13	up	glucose-6-phosphate 1-dehydrogenase (predicted)
SPBC8E4.03	2.23	up	agmatinase 2 (predicted)
SPAC3A11.07	0.48	down	NADH dehydrogenase
**Cellular inorganic cation homeostasis/iron ion homeostasis/iron assimilation by reduction and inorganic cation and transition metal ion transport**			
fip1	2.24	up	iron permease Fip1
str3	2.17	up	siderophore-iron transporter Str3 (PMID 12888492)
fio1	2.25	up	iron transport multicopper oxidase Fio1
cad1	0.39	down	sulfide-quinone oxidoreductase
**Others**			
aes1	3.40	up	enhancer of RNA-mediated gene silencing (PMID 12034844)
ipk1	2.49	up	inositol 1,3,4,5,6-pentakisphosphate (IP5) kinase (PMID 10960485)
inv1	7.94	up	beta-fructofuranosidase
mug146	2.86	up	meiotically upregulated gene Mug46
hpm1	4.25	up	homologous Pmf1p factor 1
SPCC550.07	3.72	up	acetamidase (predicted)
SPAC869.01	2.29	up	amidase (predicted)
SPBPB2B2.05	2.92	up	GMP synthase [glutamine-hydrolyzing] (predicted)
SPAC1039.08	2.25	up	serine acetyltransferase (predicted)
SPAC1002.17c	3.46	up	uracil phosphoribosyltransferase (predicted)
SPAC4F10.17	2.08	up	conserved fungal protein
SPCC584.16c	3.23	up	sequence orphan
SPAC6C3.03c	2.58	up	sequence orphan
SPBPB7E8.02	2.64	up	conserved protein (fungal bacterial protazoan)
urg1	5.06	up	GTP cyclohydrolase (predicted)
tlh2	0.42	down	RecQ type DNA helicase Tlh1
SPAC1786.02	0.49	down	phospholipase (predicted)
SPBPB2B2.08	0.46	down	conserved fungal protein
SPAC750.03c///SPAC977.03	0.48	down	methyltransferase///methyltransferase (predicted)
///SPBC1348.04			///methyltransferase (predicted)
SPAC26F1.11	0.37	down	sequence orphan
SPAPB1A10.14	0.41	down	F-box protein, unnamed
SPAC2H10.01	0.34	down	transcription factor
SPBPB21E7.08	2.56	up	unknown

**Table 2:** Confirmation of microarray data by real-time PCR.

Gene symbol	Microarray		Real-time PCR	
	Fold change	Regulation	Fold change	Regulation
inv1	7.94	up	2.31	up
mug146	2.86	up	2.70	up
psi	2.12	up	3.46	up
SPCC550.07	3.72	up	9.01	up
caf1	1.40	down	1.68	down
cad1	2.53	down	7.63	down
pyp1	1.80	down	1.55	down
rrp8	1.60	down	1.67	down

### Functional classification of differentially expressed genes in response to H_2_S

We analyzed the functional classification of differentially expressed genes that changed by greater than 1.5 or 2 fold by DAVID [21]. The analysis of genes whose expression level changed greater than 1.5 fold are shown in Table S1. Table 1 shows the 80 differentially expressed genes (greater than 2 fold) classified into known or predicted functional groups. These genes encode proteins that are involved in the following cellular functions. (1) Thirty one genes are known or predicted to function in stress responses. The details of comparison of H_2_S response to stress response are described in the next section. (2) Sixteen genes encode putative or known membrane transporters including carbohydrate/monosaccharide transporters (ght1, ght3, ght4, ght5 and ght6), ion transporters involved in iron assimilation/homeostasis (fip1, str3 and fio1) and 3 amino acid transporters (SPBPB2B2.01, SPAP7G5.06 and isp5). Most of these are membrane transporters. H_2_S is known to effect ATP-sensitive potassium (K_ATP_) channels and other transmembrane ion transport proteins [29]. The transporters identified here are regulated by H_2_S on a transcriptional level. The mechanism and cellular function of these transporters in response to H_2_S will require further investigation. (3) Eleven genes encode proteins that are involved in cell cycle/meiosis in agreement with the observation that H_2_S can act on cell cycle related genes and apoptosis [29]. (4) Ten genes encode oxidoreductases. Interestingly, of the 2 fold down (FDR<0.05) regulated genes all 3 (gut2, cad1 and SPAC3A11.07) and 6 out of 7 of the 1.5 fold down (FDR<0.05) regulated genes (ade9, gut2, cad1, qcr7, rip1 and SPAC3A11.07) are located in the mitochondrion (Table S1). The effects of H_2_S on the transcription of mitochondrial genes and mitochondrial function are presented in the relevant section below.

Of the genes that exhibit FDR<0.05 and 1.5 fold changes, we found that in response to H_2_S, 19 genes involved in ribosomal biogenesis were all down regulated. The repression of the cellular protein synthesis machinery is consistent with the observation that cell growth is inhibited in wild-type cells treated with NaHS, and may also be a part of the cellular stress response.

### Comparison of H_2_S response with stress response

**Figure 3:**
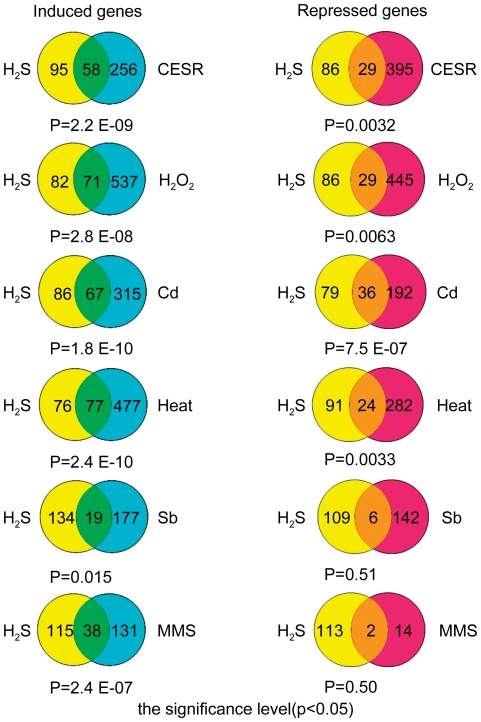
Comparison between the H_2_S response and the environmental stress response. The comparison between the genes induced or repressed more than 1.5 fold (FDR<0.05) in response to H_2_S and CESR genes are illustrated in Venn diagrams. The genes whose expression induced or repressed more than 1.5 fold in response to H_2_S and genes whose expression changed more than 2 fold in respond to H_2_O_2_, cadmium, heat, sorbitol and MMS stresses are presented in Venn diagrams. The numbers of the overlapping genes are illustrated in Venn diagrams and the gene list is available in the Table S2. Fisher test is used to evaluate the statistical significance of overlaps between two gene groups and p value is placed for each Venn diagram. For the overlaps to be statistical significant, the p value should be <0.05.

From our functional classification analysis of differentially expressed genes that respond to NaHS treatment, 31 of 80 genes whose expression changed more than 2 fold (FDR<0.05) are also stress response genes, indicating a close relationship between the stress response and the cellular response to H_2_S. We next examine in detail the stress response and H_2_S response in fission yeast. The global gene expression response of fission yeast to 5 different environmental stresses (hydrogen peroxide, cadmium, heat, sorbitol and MMS) has been characterized by microarray [18]. The core environmental stress response (CESR) genes were defined as genes whose expression changes most in response to the stresses. H_2_S may also be regarded as a form of cellular stress. To investigate similarities between the H_2_S response and the stress response, we compared genes whose expression changed 1.5 fold (FDR<0.05) in response to H_2_S with the CESR genes and genes whose expression changed 2 fold in response to the 5 different environmental stresses. The numbers of overlapping genes between these groups are shown in the Venn diagram (Figure 3) and overlapping groups listed (Table S2). We used the Fisher test to evaluate the statistical significance of overlap between the two groups of genes. There is considerable overlap between genes induced by H_2_S and CESR genes (P value<0.01) or genes that are induced in hydrogen peroxide (P value<0.01), cadmium (P value<0.01), heat shock (P value<0.01), sorbitol (P value<0.05) and MMS (P value<0.01). However, there are differences in the overlap between genes repressed by H_2_S and CESR genes that are repressed by hydrogen peroxide, cadmium, heat shock, sorbitol and MMS; characterized by P values<0.05 except for MMS (with a P value of 0.51). This indicates that the overlap between the H_2_S repressed genes and MMS repressed genes is not statistically significant. This analysis shows that 46% of H_2_S induced genes were also induced by hydrogen peroxide, 44% by cadmium and 50% by heat but only 12% and 25% of H_2_S induced genes were induced by sorbitol and MMS respectively. It has been suggested that H_2_S protects cells from oxidative stress in general [30]. Our data showed that 46% of H_2_S induced genes were induced by hydrogen peroxide and would explain why H_2_S protects cells from oxidative stress. In fission yeast, Sty1 is a key protein kinase in the MAPK pathway in stress response. The MAPK protein kinase cascade in fission yeast is homologous to that in other model organisms and humans. The Sty1 down-stream target genes were identified by genome-wide analysis in response to stress using a Sty1 deletion strain in fission yeast [18]. The components of the MAPK pathway ERK and p38 have been reported to respond to H_2_S exposure [29] and in corroboration of this; we note that a number of H_2_S induced genes are targets of the MAPK pathway (Table 3). The molecular mechanism for the effects of H_2_S on the MAPK pathway is not known. The target genes of the MAPK pathway identified here in response to H_2_S may provide useful clues.

**Table 3:** Sty1 stress response genes showing a greater than 1.5-fold induction in response to H2S.

Gene symbol	Fold change	Gene annotation
SPBP4H10.10	2.53	rhomboid family protease
SPAC27D7.09c	2.38	S. pombe specific But2 family protein
SPCC191.01	2.16	sequence orphan
SPAC4H3.03c	1.90	glucan 1,4-alpha-glucosidase (predicted)
SPAC27D7.09c///SPAC27D7.10c	1.83	S. pombe specific But2 family protein///S. pombe specific But2 family protein
SPAC2E1P3.01	1.73	zinc binding dehydrogenase
etr1	1.70	enoyl-[acyl-carrier protein] reductase
SPAC977.13c	1.61	unknown
SPCC576.04	1.58	bax inhibitor-like protein
isp6	1.50	vacuolar serine protease Isp6
SPAC57A7.05	1.48	conserved protein (fungal and plant)
ish1	1.46	LEA domain protein

In budding yeast a correlation has been noted between the environmental stress response genes and genes whose expression also correlates to growth rate [31]–[33], suggesting that for such genes the observed response to stress is secondary to a specific effect of the imposed stress on growth rate as has been observed recently in the Atf1/Pcr1 transcriptional response to oxidative stress in fission yeast [34]. Some transcription factors have been shown to be involved in the effect of H_2_S on cells [1]. We have identified 7 putative transcription factors (SPCC320.03, mts2, SPCC1223.13, scr1, SPAC2H10.01, SPBC530.11c and SPCC1393.08) that are differentially expressed (Table S1) in response to H_2_S in this study. Although analogous growth rate data are not available for fission yeast, the similarities in the stress response between the two yeasts has been documented [18]. We therefore compared the differentially expressed genes that display a significant overlap between effects of H_2_S and the stress response with their orthologues that have a correlation between growth rate and the ESR in budding yeast [33]. Interestingly, of the 58 CESR genes that were induced by H_2_S, 10 (corresponding to P value of <0.05) had been identified as having both growth rate and stress dependence in budding yeast (Table S2) suggesting that for this subset of genes the H_2_S/stress response that we observe may be due to a secondary stress effect on cell growth. In contrast, only one of the 29 CESR genes (corresponding to P value of 1) that were repressed by H_2_S was growth rate and stress dependent in budding yeast (Table S2).

### Effect of H_2_S on the transcription of mitochondrial genes and mitochondrial function

**Figure 4:**
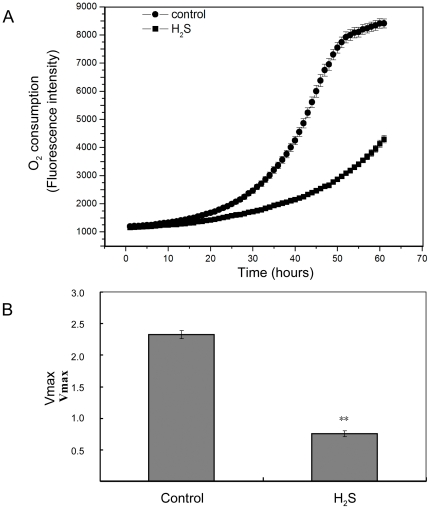
Oxygen consumption in S. *pombe* in response to H_2_S. Equal volumes of cells were separated into aliquots in wells of a 96-well BD Oxygen Biosensor plate. Three technical repeats were performed. Plates were covered and the fluorescence of each well was recorded over time with a fluorescence microplate spectrophotometer. A. oxygen consumption curves. Values are means ± SD of the results from three technical repeats. This experiment also has three biological repeats. B. quantitative changes in the respiratory rate of *S. pombe* cells were calculated from kinetic measurements. Vmax is the maximum oxygen consumption rate. Values are mean ±SD of the results from three biological experiments; **P<0.01 versus untreated group (control).

A genome-wide protein localization study using GFP fusion proteins has experimentally localized all of the proteins in fission yeast [35]. Based on this genome-wide protein localization data, we identified the sites of protein localization of the genes that display a greater than 1.5 fold change in transcription level in response to H_2_S. This analysis shows the repressed genes to be enriched for proteins that were experimentally localized to the mitochondrion (Table S1). In the genome-wide protein localization study, 9.7% of genes were found to be localized in the mitochondrion (480 genes out of 4954 genes) [35]. Of the genes that were repressed upon NaHS treatment by greater than 1.5 fold, 20% were found to localize to the mitochondrion (23 of 115 genes). This enrichment is significant and suggests that upon H_2_S treatment expression of many mitochondrial genes is reduced and this may have an impact on mitochondrial function. The 23 genes encode mitochondrial proteins that are part of the respiration machinery including Gut2 (glycerol-3-phosphate dehydrogenase), SPAC3A11.07 (NADH dehydrogenase), rip1 (ubiquinol-cytochrome-c reductase complex subunit 5), cox13 (cytochrome c oxidase subunit VIA). We also note that certain mitochondrial genes are an essential component for cellular survival upon extrinsic oxidative stress [36], and speculate that the changes in gene expression of the mitochondrial proteins in response to H_2_S that we observe may affect mitochondrial respiratory function. To investigate this we used a fluorescence based assay to investigate the changes in the mitochondrial oxygen consumption in response to NaHS treatment. Cells treated with or without NaHS were placed in a 96-well Oxygen Biosensor plate. The bottom of the plate is embedded with an oxygen-sensitive dye. Oxygen in the media quenches the ability of the dye to fluoresce. When cells grow in the well, the concentration of oxygen is reduced to allow the dye to fluoresce. The amount of fluorescence measured correlates directly to the rate of oxygen consumption. The fluorescence signal in each well was recorded over time with a fluorescence microplate spectrophotometer. Figure 4A is an oxygen consumption curve of *S. pombe* cells with or without treatment of NaHS. Data in Figure 4 shows that mitochondrial oxygen consumption was significantly reduced in cells treated with NaHS. This result is consistent with our observation that the transcription of many of the mitochondrial genes that are involved in mitochondrial respiration was reduced when cells are treated with NaHS. Our data suggests that H_2_S reduces mitochondrial respiration at the transcriptional level through the down-regulation of the components and subsequent disruption of the mitochondrial respiratory machinery. The mechanism by which H_2_S affects the expression of mitochondrial respiratory proteins at the transcriptional level is intriguing and requires further investigation. It is known that H_2_S can inhibit cytochrome c oxidase and reduce ATP production in the mitochondrion. Interestingly, we found that in response to H_2_S most genes that encode the cytochrome c oxidase subunit were slightly down regulated and cox13 is 1.8 fold down regulated. Several cytochrome c reductases were reduced (Table 4). Our data therefore suggests that H_2_S may inhibit cytochrome c oxidase on a transcriptional level.

**Table 4:** Transcription profile of genes that encode components of mitochondrial respiration in response to H_2_S.

Gene symbol	Fold change	Regulation	Gene annotation
cox5	1.39	down	cytochrome c oxidase subunit V
cox6	1.29	down	cytochrome c oxidase subunit VI
cox13	1.80	down	cytochrome c oxidase subunit VIa
cyt1	1.43	down	cytochrome c1
mas1	1.64	down	mitochondrial processing peptidase complex beta subunit Qcr1
qcr2	1.32	down	ubiquinol-cytochrome-c reductase complex core protein Qcr2
qcr6	1.22	down	ubiquinol-cytochrome-c reductase complex subunit 8
qcr7	1.51	down	ubiquinol-cytochrome-c reductase complex subunit 6
qcr8	1.43	down	ubiquinol-cytochrome-c reductase complex subunit 7
qcr9	1.32	down	ubiquinol-cytochrome-c reductase complex subunit 10
rip1	1.87	down	ubiquinol-cytochrome-c reductase complex subunit 5

## Supporting Information

Table S1 Genes that display a 1.5 fold change in expression level (SAM analysis FDR<0.05) in response to H_2_S.
(XLS)

Table S2 Overlap between genes that display a 1.5 fold change (FDR<0.05) in expression in response to H_2_S with the cellular environmental stress response genes and genes whose expression changed 2 fold in response to the 5 different environmental stresses.
(XLS)
